# Reconstructing codependent cellular cross-talk in lung adenocarcinoma using REMI

**DOI:** 10.1126/sciadv.abi4757

**Published:** 2022-03-18

**Authors:** Alice Yu, Yuanyuan Li, Irene Li, Michael G. Ozawa, Christine Yeh, Aaron E. Chiou, Winston L. Trope, Jonathan Taylor, Joseph Shrager, Sylvia K. Plevritis

**Affiliations:** 1 Department of Biomedical Data Science, Stanford University, Stanford, CA, USA.; 2 Department of Radiology, Stanford University, Stanford, CA, USA.; 3 Cancer Biology Interdepartmental, Program Stanford University, Stanford, CA, USA.; 4 Department of Pathology, Stanford University, Stanford, CA, USA.; 5 Department of Cardiothoracic Surgery, Stanford University, Stanford, CA, USA.; 6 Department of Statistics, Stanford University, Stanford, CA, USA.

## Abstract

Cellular cross-talk in tissue microenvironments is fundamental to normal and pathological biological processes. Global assessment of cell-cell interactions (CCIs) is not yet technically feasible, but computational efforts to reconstruct these interactions have been proposed. Current computational approaches that identify CCI often make the simplifying assumption that pairwise interactions are independent of one another, which can lead to reduced accuracy. We present REMI (REgularized Microenvironment Interactome), a graph-based algorithm that predicts ligand-receptor (LR) interactions by accounting for LR dependencies on high-dimensional, small–sample size datasets. We apply REMI to reconstruct the human lung adenocarcinoma (LUAD) interactome from a bulk flow-sorted RNA sequencing dataset, then leverage single-cell transcriptomics data to increase the cell type resolution and identify LR prognostic signatures among tumor-stroma-immune subpopulations. We experimentally confirmed colocalization of CTGF:LRP6 among malignant cell subtypes as an interaction predicted to be associated with LUAD progression. Our work presents a computational approach to reconstruct interactomes and identify clinically relevant CCIs.

## Teaser

Biocomputational can reconstruct the multicellular interactome by accounting for ligand-receptor interdependency.

## INTRODUCTION

Cell-cell interactions between and within the various cell types comprising the tissue microenvironment play a fundamental role in regulating local and systemic biological and physiological functions under normal and pathological conditions. These interactions facilitate cooperation or competition between cell types and are typically mediated between ligands and receptors. Ligands are often manifested as soluble or extracellular proteins that are expressed by the “sending” cells and bind onto a cognate receptor on the “receiving” cells (*1*, *2*). In tumor microenvironments (TMEs), cellular cross-talk between tumor, stroma, and immune cells orchestrates the establishment of preinvasive and invasive niches that enable cancer progression properties, such as tumor growth, immune evasion, and metastasis. Large-scale cellular interactions are difficult to measure using current experimental techniques, but several computational approaches have been proposed to predict these interactions using -omics data.

A majority of the current computational approaches that use high-throughput transcriptomics data to infer cell-cell interactions either calculate interaction scores on the basis of gene expression permutation tests or implement graph-based approaches (*2*). In expression permutation-based approaches, such as CellphoneDB v2.0 and NATMI, potential ligand and receptor (LR) interactions are identified by thresholding the genes on the basis of their expression level with the assumption that this predicts higher LR protein abundance (*3*, *4*). Other methods, such as CCCExplorer and NicheNet, calculate correlation metrics between the expression levels of the ligand, receptor, or downstream signaling pathway genes for each LR pair (*5*, *6*). However, a correlation between the expression of ligand, receptor, or downstream genes may be capturing an indirect association caused by another LR interaction involving the ligand or receptor of interest.

While current cross-talk inference approaches provide a valuable baseline for synthesizing hypotheses of LR interactions, they do not capture the conditional dependencies of LR pairs among multiple cell types. Current approaches make the assumption that pairwise interactions are independent, but a pairwise interaction can be influenced by other interactions via autocrine and paracrine loops (*7*, *8*). Calculating the conditional dependency of LR pairs is often an ill-defined problem for high-dimensional datasets generated across a small sample size. This challenge is common in -omics data analysis because of the large number of parameters (*p*) and relatively small number of samples (*n*). To address this challenge, we present the algorithm, called REMI (REgularized Microenvironment Interactome), to identify communities of dependent LR pairs in high-dimensional datasets of small sample size using graph-based approaches. We demonstrate the performance of REMI by simulating datasets with varying sample sizes to show how REMI outperforms existing approaches.

To compare REMI to existing interactomes, we focused on reconstructing the lung adenocarcinoma (LUAD) interactome. Various renditions of the LUAD microenvironment have been assembled using different computational approaches that have led to novel insights. Kumar *et al.* (*9*) built an interactome for LUAD using mouse single-cell RNA sequencing (RNA-seq) (scRNA-seq) data, where they used a scoring mechanism that captured highly expressed LR genes. Gentles *et al.* (*10*) created the Lung Tumor Microenvironment Interactome (LTMI) from bulk flow-sorted RNA-seq data by thresholding gene expression levels in the dataset and computing pairwise correlations between LR genes. We applied REMI to the LTMI dataset to reconstruct a rendition of LUAD interactome (REMI-LUAD) with higher specificity. We then projected an independent LUAD scRNA-seq dataset onto REMI-LUAD to increase the cell type resolution of the interactome and referred to this interactome as the single-cell rendition of REMI-LUAD (scREMI-LUAD). Using scREMI-LUAD, we identified paracrine interactions between cell subtypes that were previously annotated as autocrine signaling interactions. To derive a signature of LUAD progression from scREMI-LUAD, we assigned a prognostic score to each cell subtype and identified prognostically associated cross-talk signatures that may lead to clinically relevant biomarkers and therapeutic targets. In summary, REMI offers a new approach to infer cell-cell interactions between many cell types by accounting for conditional dependencies in cell-cell interactions. REMI is implemented in R and is freely available on GitHub (https://github.com/plevritis-lab/REMI).

## RESULTS

### Impact of correlation versus partial correlation analysis on LR pair inference

**Fig. 1.**
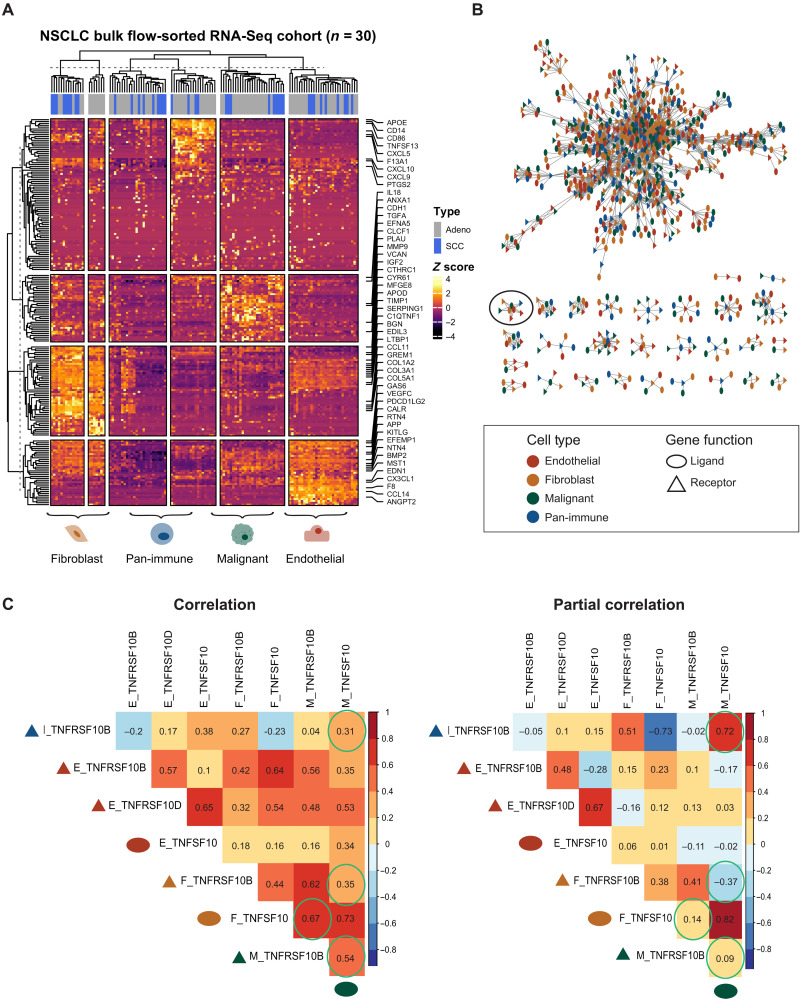
Network representation of LTMI shows dense components of cognate LR pairs. (**A**) All LR genes in the LUAD flow-sorted RNA-seq dataset hierarchically clustered. Genes labeled are the most highly expressed genes in the top five percentile. (**B**) Correlation network of expressed LR pairs from the Gentles *et al.* dataset. Color of nodes represent cell type of gene. Shape of node represents type of gene. Black circle indicates network component used for correlation analysis in panel below. (**C**) Correlation and partial correlation values of the nodes that are found within the black circled component in (B). Correlation matrix values are on the left, and partial correlation matrix values are on the right. The text labels of the correlation matrices correspond to the gene’s cell type added in front of the gene name (celltype_name). In addition, a colored (cell type) shape (gene function) is placed in front of the text label. Colors correspond to the legend in (B). Bottom dark green circle corresponds to M_TNFSF10 labeled at the top of the column. Green circles highlight examples of correlation values that are changed after accounting for confounding variables.

Many of the current computational approaches use correlation to identify likely LR interactions. Here, we show that correlation-based analyses can provide high false-positive predictions that are removed when computing the partial correlation. Partial correlation removes potential confounding effects from correlation values that are caused by another ligand or receptor indirectly affecting the expression levels of the LR pair of interest. To demonstrate this point, we analyzed a publicly available primary LUAD bulk flow-sorted RNA-seq dataset, which contains transcriptomic data for four main cell types: malignant, fibroblast, endothelial, and pan-immune cells (GSE111907) (*n* = 17 patients) (*10*). As expected, hierarchical clustering performed on only the genes that expressed ligands and receptors grouped the samples by cell type (Fig. 1A). We then constructed an LR correlation network, denoted as *G*_LTMI − LUAD_, where the nodes represent ligand or receptor genes, and an edge between an LR pair exists on the basis of the threshold criteria set by Gentles *et al.* (Fig. 1B). The edge weight is set as the Pearson correlation between the gene expression of the LR.

Because many receptors have multiple potential ligand pairings and vice versa, the correlation network is composed of a large dense network with small disjoint network components, as indicated in Fig. 1B. To infer the conditional dependency of LR pairs within the network, we calculated the partial correlation of genes on a small disjoint graph, which contains one ligand node (*TNFSF10)* and two receptor nodes (*TNFRSF10B* and *TNFRSF10D)* across multiple cell types (Fig. 1B). Although simultaneous TNFSF10:TNFRSF10B and TNFSF10:TNFRSF10D interactions could occur, one interaction plays an agonistic role, while the other plays an antagonistic role (*11*). The binding of TNFSF10 to TNFRSF10B induces cell apoptosis via the TRAIL [tumor necrosis factor (TNF)–related apoptosis-inducing ligand] pathway, whereas the binding of TNFSF10 to TNFRSF10D inhibits the TRAIL pathway (*12*). If we infer LR interactions on the basis of correlation, then the ligand TNFSF10 secreted from the malignant cell interacts with the receptor TNFRSF10B on malignant and fibroblast cells (circled in green), indicating likely apoptosis of malignant cells and cancer-associated fibroblasts (Fig. 1C, left). This contradicts studies that show protumoral effects of fibroblasts and how they can express the decoy receptor to avoid apoptosis (*13*). Instead, by calculating the partial correlation of the LR pairs within the circled component, we find more biologically reasonable results. On the basis of the partial correlation values, TNFSF10 expressed by the malignant cells is more likely to interact with TNFRSF10B expressed on the immune cells, inducing protumoral effects as described in literature (*12*, *14*). This simple analysis demonstrates how partial correlation removes confounding effects in correlation analysis that may be misleading by reducing positive edges when building the interactome (Fig. 1C, right). However, calculating the partial correlation is ill-posed on larger subnetworks because of the high dimensionality of many -omics datasets. REMI extends this concept onto larger components of the network.

### REMI algorithm

**Fig. 2.**
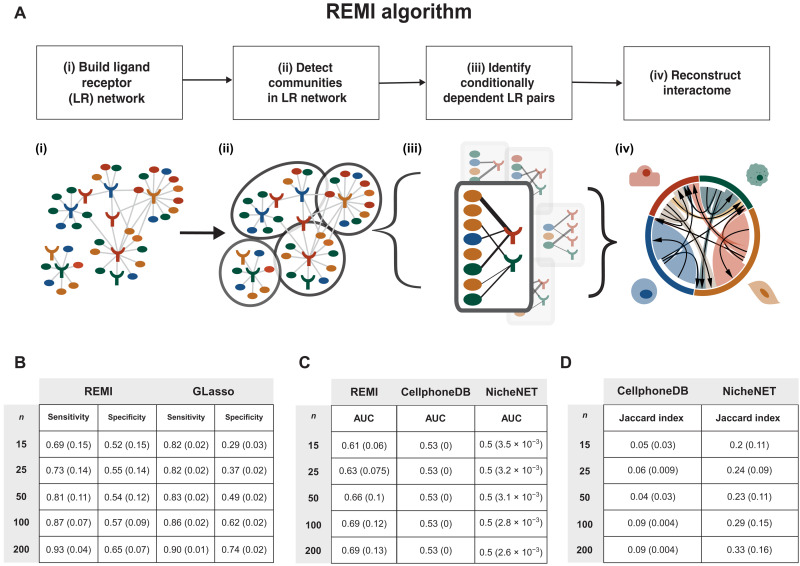
Schematic overview of REMI and simulation studies. (**A**) Diagram of REMI: (i) A network of potential LR interactions between cell types of interest is built given a reference LR database. Nodes represent either ligand or receptor genes. Edges represent known LR pairs. (ii) Network represented as density-based communities. (iii) Conditionally independent LR pairs removed: Each community is converted into a graphical Gaussian model, and conditionally independent LR pairs are removed. (iv) Conditionally dependent LR pairs across communities are reassembled into global interactome. (**B**) Simulation studies: Performance of REMI versus GLasso for sample sizes *n* = 15 to 200 when compared to a GLasso interactome derived from the entire LUAD TCGA RNA-seq dataset (*n* = 1031). Cohorts of various sample sizes were generated by downsampling TCGA data and then used to compare REMI’s and GLasso’s performance. At each sample size, 50 cohorts were generated. Mean AUC and SD (in parentheses) values are generated across the 50 cohorts per sample size. (**C**) Test performance of REMI compared with other computational cross-talk prediction algorithms. At each sample size, 50 cohorts were generated by randomly sampling TCGA data. Mean AUC and SD (in parentheses) are provided. (**D**) Average Jaccard indices of 50 cohorts per sample size calculated for each method compared to REMI.

We introduce our novel algorithm, REMI, which identifies communities of conditionally dependent cell-cell interactions to reduce the number of false-positive edges in LR correlation–based networks. The algorithm is applicable to high-dimensional transcriptomic datasets with small sample sizes. REMI is composed of four steps: (i) build a weighted undirected LR correlation network leveraging known LR pairings, (ii) detect communities of LR groups, (iii) identify conditionally dependent LR pairs in communities, and (iv) reconstruct the global interactome from the communities (Fig. 2A) (Materials and Methods). An additional step in REMI allows for the user to measure the significance of an LR pair prediction with respect to the LR pair’s REMI community.

In its first step, REMI generates a weighted bipartite LR network, denoted by *G*, where the nodes represent either a ligand or a receptor gene expressed in a specified cell type within the dataset. Edges of *G* are drawn between literature-supported LR pairings curated from the FANTOM5 database (*15*). Edge weights of *G* are computed as the Pearson correlation between the gene expression of the LR nodes (Fig. 2, A and I). Ideally, the conditional dependency of each LR pair would be computed with respect to all other nodes in the network. However, current high-dimensional transcriptomic datasets contain a large number of genes compared to the number of samples, which makes the conditional dependency hard to estimate with high accuracy.

To reduce the dimensionality of the problem, REMI hierarchically divides *G* into groups of densely connected nodes, called communities, using the Louvain community detection method (*16*). Next, REMI identifies conditionally dependent LR pairs in each community independently. A covariance matrix between all pairs of ligands and receptors is computed for each community, setting edges between ligands and edges between receptors as zero (Fig. 2A, ii). To identify these conditionally dependent LR edges within a community, we use graphical lasso (GLasso), which estimates the inverse covariance of the community using a lasso penalty that increases the graph’s sparsity (Fig. 2A, iii) (*17*). The resulting edge weights represent the *l*1-regularized partial correlation metrics of each LR pair within a community, which captures its relationship with respect to other LR pairs in the community. For further downstream analyses, we analyzed a binarized version of the network, denoted as $\hat{\Theta }$, reconstructed by aggregating the communities after filtering for edges with a positive edge weight.

REMI’s iterative community detection algorithm component allows for community sizes to change for downstream prediction. We ran several simulations to test the sensitivity and specificity of varying community sizes. REMI’s best performance occurs when the number of nodes in each community is equal to the sample size (fig. S2B). Therefore, REMI hierarchically breaks down large communities until the size of the communities is approximately equivalent to sample size. Communities identified by Louvain are referred to as “within communities” (WC). This step prioritizes assigning a node to a unique community but leaves edges that represent potential LR pairs unassigned. REMI creates additional communities to account for the newly unassigned edges by aggregating unassigned edges that exist between two communities to create “between communities” (BCs) (fig. S1A). The conditionally dependent WC and BC edges are aggregated to reconstruct the interactome (Fig. 2A, iv).

To reconstruct the global interactome from the independent community calculations, we developed a method to measure the significance of each computed edge with respect to its community. The *P* value is calculated by randomly permuting the edge weight of the LR pair with randomly sampled correlation values. Because multiple correlation values can predict a binary edge in REMI, we build a decision tree to sort out which correlation values predict the edge of interest. We then sample from our null Wishart distribution, which represents a previous covariance matrix distribution, and predict edges using the decision tree. Edges predicted using values sampled from the null represent our REMI null distribution. We then calculate a one-sided *P* value by measuring the number of null edges that had a greater correlation value than the original edge weight. The one-sided *P* value is then converted to a two-sided *P* value (fig. S1B). Although the statistical test is computationally intensive, it can be used for determining the presence or absence of the edges when aggregating the communities to reconstruct the global interactome.

### Testing robustness of REMI parameters via simulations

To assess REMI’s performance with respect to implementing GLasso alone, we generated a population-level regularized interactome by leveraging the large publicly available Cancer Genome Atlas [The Cancer Genome Atlas (TCGA)] LUAD bulk RNA-seq dataset. We made the assumption that the 1013 patients in this dataset represent an entire population and regularized a network of LR pairs within the dataset using GLasso. Because of the large sample size, confounding effects for all possible interactions within the microenvironment are captured, and the resulting network, denoted as ${\hat{\Theta }}_{\text{TCGA}}$ (Supplementary Dataset), contains these conditionally dependent LR pairs.

From the TCGA LUAD cohort, we created randomly subsampled cohorts of different sample sizes (*n* = 15, 25, 50, 100, and 200), generating 50 cohorts at each sample size. We ran REMI and GLasso on each downsampled cohort and compared their performance in terms of sensitivity and specificity (Fig. 2, B and C). We found that in larger sample sizes (*n* = 200), REMI and GLasso performed with comparable sensitivity (0.93 and 0.90, respectively) and specificity (0.65 and 0.74, respectively) (Fig. 2B). As the sample size decreased (*n* = 15), GLasso retained high sensitivity values compared with REMI. GLasso’s sensitivity dropped from 0.90 to 0.83, whereas REMI’s sensitivity dropped from 0.93 to 0.69. However, REMI retained higher specificity than GLasso as the sample size decreased: GLasso’s specificity dropped from 0.74 to 0.29 (45% decrease), whereas REMI’s specificity dropped from 0.65 to 0.52 (13% decrease). In summary, REMI has higher specificity compared with GLasso for datasets with smaller sample sizes, which we regard as a preferred property when selecting candidate-relevant interactions for experimental validation.

To further evaluate the performance of REMI, we ran the same simulations and measured the sensitivity and specificity of WC and BC REMI predictions as a function of sample size. WCs have a higher density compared with BCs and yielded relatively higher sensitivity and lower specificity as sample size decreased from *n* = 200 to *n* = 15. In WCs, sensitivity dropped from 0.93 to 0.75 (18% decrease), and specificity dropped from 0.63 to 0.45 (18% decrease). On the other hand, BCs performed with relatively lower sensitivity and higher specificity as sample size decreased from *n* = 200 to *n* = 15. In BCs, sensitivity dropped from 0.88 to 0.45 (43%), and specificity stayed relatively the same from 0.74 to 0.76 (2% increase) (fig. S2A). Next, we considered the effect of the community size on REMI’s performance. REMI’s community sizes are set to be equal to the sample size of the dataset. When the number of nodes within a community was increased from 2*n* to 50*n* times, the density of the communities increased and specificity decreased from 0.52 to 0.33 (19%), whereas sensitivity increased from 0.66 to 0.78 (12%) (fig. S2B). Community sizes closer to the sample size of the dataset have increased specificity as opposed to larger community sizes, thereby reinforcing REMI’s optimization for specificity.

Next, we compared REMI to current cell-cell interaction computational inference approaches designed for bulk and scRNA-seq. We selected methods that represent the expression permutation-based approaches (CellphoneDB v2.0) and network-based approaches (NicheNet) and ran the methods on the same simulation (Fig. 2C). REMI’s performance ranged from an area under the curve (AUC) of 0.61 to 0.69 as sample size increased. CellphoneDB v2.0 performed with an AUC of 0.53, and NicheNet performed with an AUC of 0.5 across all cohort sizes. Both methods’ performance did not vary as size increased, suggesting that these approaches are more biased toward the reference LR network and do not readily adapt to the variations seen in the data. We also calculated the Jaccard index of NicheNet and CellphoneDB v2.0’s results compared to REMI and found both to be low because of the high number of predicted interactions in NicheNet and CellphoneDB v2.0. NicheNet’s regression-based approach had higher Jaccard indices (0.20 to 0.33) as opposed to CellphoneDB v2.0 (0.05 to 0.09), showing more similarity between NicheNet and REMI rather than CellphoneDB v2.0 and REMI.

### Reconstructing LUAD interactome using REMI

**Fig. 3.**
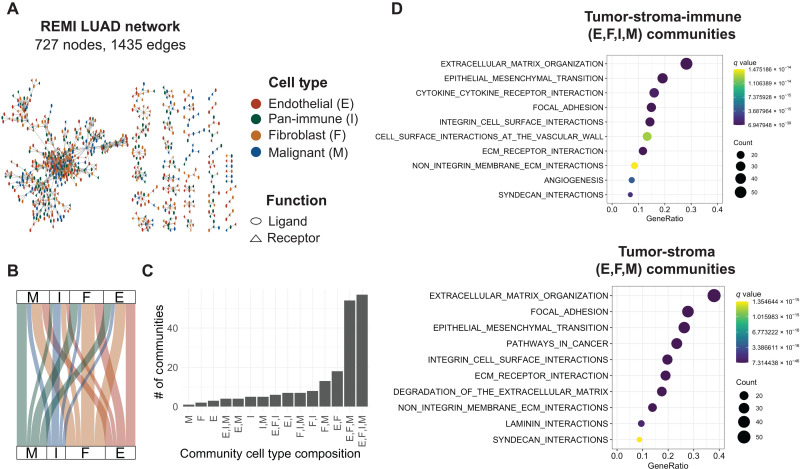
Reconstructed LUAD TME interactome reveals tumor-stroma–specific communities. (**A**) Force-directed layout of the REMI-LUAD interactome network. Shape of the nodes represent gene function. Color of the node represents cell type as denoted by the legend. (**B**) Alluvial plot showing the ratio of paracrine and autocrine signaling interactions occurring between cell types. Thickness of lines represent the number of LR pairs. Full table is available in the Supplementary Materials. (**C**) Distribution of number of communities with certain cell type compositions. (**D**) Top 10 most significant enrichments for genes within the tumor-immune-stroma–specific communities and tumor-stroma–specific communities. GeneRatio is the ratio of the number of community-specific genes that overlapped with the gene set to the number of community-specific genes that overlapped with all the gene sets in the collection. Count represents the number of genes.

We applied REMI to the bulk flow-sorted LUAD RNA-seq cohort to reconstruct the REMI-LUAD interactome, with the graph denoted as *G*_REMI − LUAD_. Compared with G_LTMI − LUAD_, *G*_REMI − LUAD_ had 16% fewer nodes (*V* = 868 in LTMI and *V* = 727 in REMI) and 45% fewer edges (*E* = 2652 in LTMI and *E* = 1435 in REMI), highlighting the regularization underlying REMI’s approach (Fig. 3A and Supplementary Dataset). REMI-LUAD removed LR edges that were densely connected (average node degree of edges removed = 12) and also identified a few new LR pairs (fig. S1C). In REMI-LUAD, known lung cancer–specific LR pairs were identified, including *GREM1:KDR*, which was experimentally validated between fibroblast and malignant cells (*10*). We observed fewer immune interactions within REMI-LUAD than within LTMI-LUAD. We suspect that this is a consequence of the greater heterogeneity among the pan-immune cells relative to the other three tumor-stroma cell types in the bulk-sorted samples, as demonstrated in published scRNA-seq studies (*18*). Grouping the diverse immune-suppressive and immune-reactive TME immune cells into a pan-immune group reduced their overall effect in REMI. We are reassured by this finding because it would have been challenging to interpret interactions of pan-immune cells and other cell types that included both tumor-promoting and tumor-suppressing features.

To understand which cell types are interacting in REMI-LUAD, we analyzed the cell type composition within each community. Majority of the communities were either represented by all cell types (E, F, I, and M) (36%) or tumor-stroma cell types (E, F, and M) (25%) (Fig. 3C). To assess the phenotypic properties enriched in these immune-stroma and stroma communities, we performed Gene Set Enrichment Analysis (GSEA) on the LR genes in each community separately. Communities with tumor-immune-stroma interactions were enriched for cytokines, angiogenesis, extracellular matrix (ECM) organization, and leukocyte extravasation. Angiogenesis and ECM remodeling are crucial for building a niche architecture for tumor growth. Communities with tumor-stroma–specific interactions were uniquely enriched for ECM degradation (Fig. 3D). ECM stiffness is associated with increased immunosuppression and found to stimulate epithelial-to-mesenchymal transformation in the tumor, whereas ECM degradation aids in creating paths for cancer cell migration (*19*). Integrins found within REMI-LUAD, in particular, can detect ECM mechanical stiffness and assist with cell migration through degraded areas (*20*). Together, our results suggest that REMI-LUAD captures cell-cell interactions associated with processes ranging from tumor growth to invasion.

### Increasing cell type resolution of REMI-LUAD using a scRNA-seq LUAD dataset

**Fig. 4.**
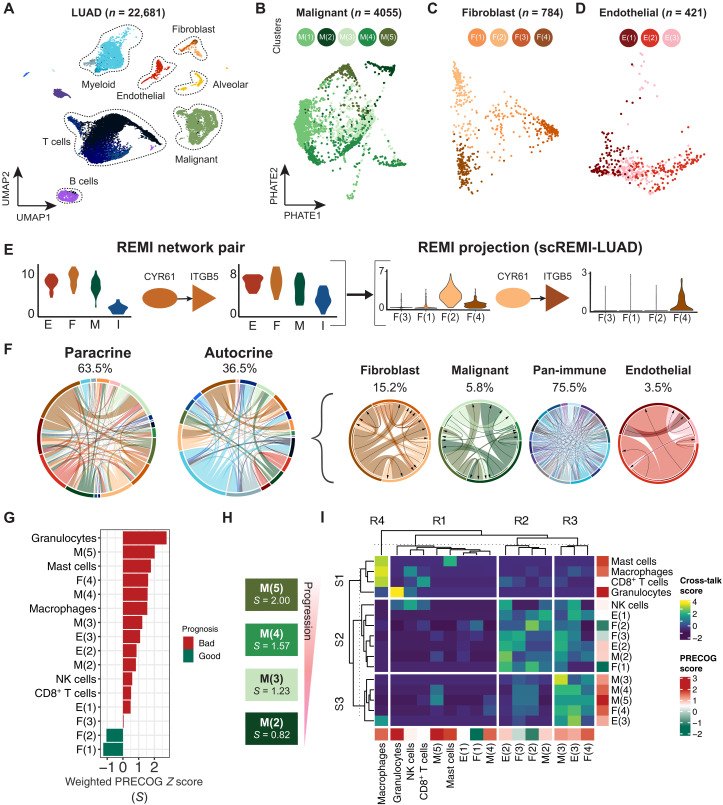
Projecting scRNA-seq information onto LUAD interactome reveals prognostic progression in malignant subpopulations. (**A**) Uniform manifold approximation and projection (UMAP) of Lambrecht *et al.* lung carcinoma scRNA-seq dataset (*n* = 22,681 cells, two patients). (**B**) PHATE (potential of heat diffusion for affinity-based transition embedding) representation of malignant cell population and its five subpopulations (*n* = 4055 cells). (**C**) PHATE representation of fibroblast population and its four subpopulations (*n* = 784 cells). (**D**) PHATE representation of endothelial population and its three subpopulations (*n* = 421 cells). (**E**) Deconvolution pipeline of REMI interactome using scRNA-seq. The example shows one LR pair where its cell type node label is relabeled by the single-cell subpopulation labels. (**F**) Proportion of interactions involving each cell type in scREMI-LUAD. Two chords on the left show interactions that were labeled as paracrine and autocrine in REMI-LUAD. Four chords on the right show that the interactions that were previously labeled as autocrine are now labeled as paracrine signaling interactions between subpopulations. (**G**) Weighted prognosis scores for each subpopulation. (**H**) Progression of malignant subpopulations ranked from poor to good prognosis. *S* indicates prognosis score value. (**I**) Clustered cell-cell interaction cross-talk scores between cell subpopulations. Cross-talk score represents the variability of the number of LR interactions between two cell types with respect to the sending cell type. Cell types are labeled by weighted prognosis score.

To increase the cell type resolution of the REMI-LUAD, we analyzed REMI-LUAD using a publicly available independent scRNA-seq LUAD dataset from Lambrecht *et al.* (*n* = 2 patients; 22,681 total cells) (E-MTAB-6149) (*21*). We reclustered them individually using Louvain clustering and applied the annotated cell type labels from the manuscript (Fig. 4A). We labeled the immune clusters with broad immune cell subtypes as denoted from the manuscript (myeloid, T cells, and B cells). For the remaining cell types, we identified five malignant [denoted as M(1), M(2), M(3), M(4), and M(5)], three endothelial [denoted as E(1), E(2), and E(3)], and four fibroblast subtypes [denoted as F(1), F(2), F(3), and F(4)] (Fig. 4, B to D, and fig. S3) (*22*).

To adapt REMI for scRNA-seq data, we averaged each gene’s expression across each cell subtype per patient. The Lambrecht *et al.* dataset consists of only two patients with LUAD, which does not provide enough power for REMI’s inverse covariance–based calculations. For this reason, we projected the scRNA-seq cell types onto *G*_REMI−LUAD_ and relabeled the nodes’ cell types on the basis of the expression levels in the single-cell dataset. This network is referred to as single-cell REMI-LUAD (scREMI-LUAD). For each subpopulation, we filtered for differentially expressed (DE) LR genes that had an averaged expression level greater than 0.4 (Fig. 4, A to C). Sixty-five percent of the interactions in REMI-LUAD were present in scREMI-LUAD, confirming the high expression of these LRs in two independent patients. Because many LR genes appear in multiple cell subtypes, the number of interactions in scREMI-LUAD is greater than in REMI-LUAD, providing potential insight into the extent of heterogeneity of the interactome. For example, REMI-LUAD inferred that fibroblasts secrete *CYR61*, which interacts with *ITGB5* on fibroblasts, suggestive of autocrine signaling. In scREMI-LUAD, *CYR61* is expressed in F(2) and *ITGB5* in F(4). Hence, the REMI-LUAD *CYR61:ITGB5* autocrine fibroblast interaction is now redefined as a paracrine signaling between different fibroblast cell subtypes in scREMI-LUAD (Fig. 4E). Notably, 86% autocrine signaling interactions were converted into paracrine interactions between cell subtypes (Fig. 4F). This increased number of paracrine interactions highlights the extent of the complex cross-talk in the TME.

### Inferring disease progression in scREMI-LUAD based on subpopulation prognostic significance

To determine which cell-cell interactions may be involved with disease progression, we computed a prognostic score for each cell subtype and a cross-talk score between pairs of cell subtypes within scREMI-LUAD. The prognostic score represents each cell subtype’s inferred prognosis based on which ligands are expressed. First, we downloaded LUAD-specific prognostic gene scores calculated from bulk survival meta-analysis from the PRECOG (PREdiction of Clinical Outcomes from Genomic profiles) database (*23*). Positive and negative PRECOG *z* scores are associated with poor and good prognosis, respectively. Then, we multiplied each DE ligand’s average cell type expression by its PRECOG score within each subpopulation. The weighted ligand scores were normalized by the sum of all the ligand’s expression levels per subpopulation (Fig. 4G). Poor-prognostic ligand scoring subpopulations included granulocytes, F(4), M(3), M(4), and M(5), and good-prognostic ligand scoring subpopulations included F(1) and F(2). The malignant subpopulations have increasingly poor-prognostic scores, suggesting that different malignant cell subpopulations may be associated with different phases of tumor progression (Fig. 4H). From this perspective, we filtered scREMI-LUAD by selecting the ligand from the cell subtype that had the closest weighted PRECOG score to its receptor to focus on interactions potentially associated with disease progression (Supplementary Dataset).

Between every cell subtype, the cross-talk score was calculated using the number of interactions occurring between each subpopulation, standardized with respect to the number of interactions involving the sending cell. This measures the variability of the number of interacting ligands for each sending cell subpopulation. Cross-talk scores were clustered using *k*-means consensus clustering, and three groups of sending (S1, S2, and S3) subpopulations and four groups of receiving (R1, R2, R3, and R4) subpopulations were identified (Fig 4I). S2 and R2 are enriched for subpopulations associated with relatively good prognosis, such as F(1), F(2), E(1), E(2), natural killer cells, and M(2). S3 and R3 are enriched for subpopulations associated with poor prognosis, including the remaining malignant subpopulations, E(3) and F(4). We highlight three clusters of scores: (i) good-prognostic interactions between S2 and R2, (ii) mixed-prognostic interactions between S2 and R3, and (iii) poor-prognostic interactions between S3 and R3. To understand what types of interactions are occurring within each group, we performed GSEA on the good-, mixed-, and poor-prognostic interactions (fig. S4). The good-prognostic interactions are enriched for coagulation and elastic fiber formation. One of the interactions occurs between SLIT2:ROBO1 as an autocrine interaction among F(2). SLIT-ROBO signaling has been shown to inhibit lung cancer migration in murine and in vitro models (*24*, *25*), and we suspect that it may play a similar role in primary lung cancer. The mixed-prognostic interactions are enriched for angiogenesis, ECM degradation, and platelet adhesion to collagen. One such interaction that occurs between M(2) and M(3), MMP7:CD151, has been confirmed to interact at the edge of LUAD nests. This suggests that mixed-prognostic interactions may occur on the invasive front (*26*). The poor-prognostic interactions were enriched for ECM degradation, angiogenesis, and Notch signaling activation. These interactions involve the ligands TNC (Tenascin-C) and VCAN (Versican), which are associated with metastatic phenotypes, such as the mesenchymal-to-epithelial transition (*27*, *28*). On the basis of the enrichments of interactions within each group, good-prognostic interactions may be associated with less invasive TMEs, poor-prognostic interactions may be associated with more invasive TMEs, and mixed-prognostic interactions may capture the transition from less to more invasive TMEs.

### In situ experimental validation of CTGF and LRP6 colocalization among malignant subpopulations in LUAD

**Fig. 5.**
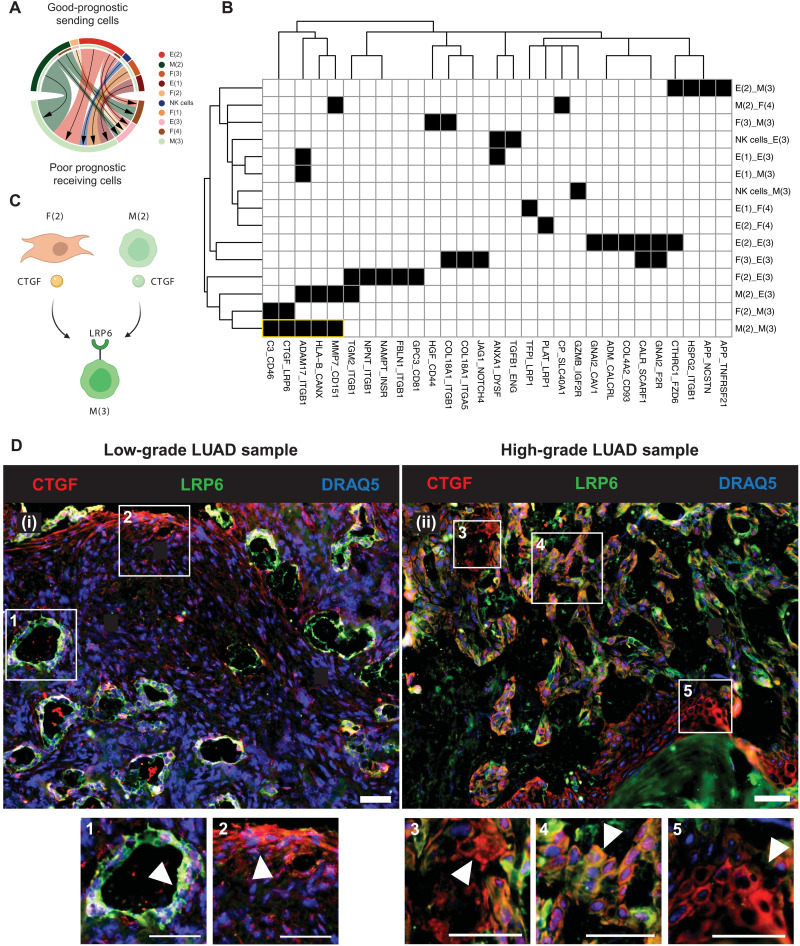
Cell-cell interaction associated with progression in LUAD. (**A**) Circle plot indicates ratio of interactions occurring in mixed-prognostic subpopulations. The color of the chords corresponds to the legend on the right. Arrows point from sending to receiving cell types. NK, natural killer. (**B**) Binarized heatmap of cell-cell interactions between good-prognostic sending subpopulations and poor-prognostic receiving subpopulations. Interactions were filtered by average receptor expression >0.5. Black indicates a predicted cell-cell interaction between LR. White indicates no LR interaction prediction. Yellow box highlights interactions occurring between M(2) and M(3). (**C**) Diagram of CTGF:LRP6 interactions predicted by scREMI-LUAD. (**D**) Immunofluorescence (IF) staining analysis of CTGF (red), LRP6 (green), and DRAQ5 (blue) in fresh-frozen LUAD samples from patients with low-grade (i) versus high-grade (ii) disease. In (i), box nos. 1 and 2 contain CTGF^−^LRP6^+^ malignant cells and CTGF^+^LRP6^−^ fibroblast cells, respectively (see H&E in fig. S6). In (ii), box nos. 3, 4, and 5 contain CTGF^+^LRP6^−^ malignant cells, CTGF^+^LRP6^+^ colocalized malignant cells, and CTGF^+^LRP6^−^ bronchial cells with potential malignancy, respectively. Scale bars, 50 μm.

We focused on the mixed-prognostic interactions because these interactions may be involved with priming the TME for invasion. Proportionally, many mixed-prognostic interactions occur between the malignant subpopulations M(2) and M(3), suggesting potential subclonal cooperation between these subpopulations (Fig. 5a). Among the M(2)-to-M(3) interactions, we highlight the LR interaction, CTGF:LRP6, because of the increasing interest in anti-CTGF therapy (Fig. 5B) (*29*, *30*). CTGF expressed by F(2) is also inferred to interact with LRP6 expressed on M(3) within the same community (*P* < 0.1) (Fig. 5C and fig. S5, A to D, H, and I). LRP6 is a coreceptor for the WNT signaling pathway and regulates tissue homeostasis (*31*). CTGF is a complex gene involved in many different processes, such as wound healing, angiogenesis, cell adhesion, migration, fibrosis, and ECM deposition (*32*). It activates both transforming growth factor–β and WNT/β-catenin downstream signaling pathways and has been found to mediate signaling during proliferative invasion, but its involvement in LUAD remains unclear (*28*).

To determine the potential phenotypic role of CTGF:LRP6, we built a downstream signaling network for malignant cells using signaling pathway genes from the Kyoto Encyclopedia of Genes and Genomes (KEGG) and protein-protein interactions from the publicly available database BioGRID (*33*, *34*). We set the edge weight as the correlation between two signaling genes calculated using the bulk flow-sorted RNA-seq dataset and measured the log-transformed eigenvector centrality score (ranging from 0 to 1) of each receptor. A low versus high score indicates that the receptor has low or high correlation, respectively, with its downstream pathway genes. This centrality score represents how influential a node is in the network in terms of how correlated it is with each of its nodes to the downstream network (fig. S5E). *LRP6*’s downstream nodes were *GSK3B* and *CTNNB1* (β-catenin) with a centrality score of 0.27 and 0.31, respectively, which is high with respect to the mean centrality score of 0.05 in the network (fig. S5, G and F). High centrality scores indicate that the genes were correlated with their signaling neighbors within the downstream network. Within the scRNA-seq dataset, M(3) cells expressed *GSK3B* and *CTNNB1*, and M(2) cells expressed *CTNNB1* (fig. S5I). The expression patterns suggest that the paracrine interaction CTGF:LRP6 may activate WNT signaling downstream within M(3) to induce an invasive phenotype. The Lrp6–Gsk3b–β-catenin–Tcf–Ctgf autocrine axis was implicated in a study of lung cancer progression in an in vivo preclinical model (*35*).

To confirm whether the colocalization of CTGF:LRP6 occurs in primary LUAD, we performed immunofluorescence (IF) and paired hematoxylin and eosin (H&E) imaging of CTGF and LRP6 in whole-tissue specimens from four patients, composed of two patients with low-grade LUAD and two with high-grade LUAD (fig. S6). Low-grade versus high-grade samples were selected because they are typically indicative of better or worse prognosis, respectively. In both low-grade samples, we see CTGF^+^ and LRP6^+^ cell subtypes, but we generally do not observe CTGF^+^LRP6^+^ colocalization [Fig. 5D (i) and fig. S6, A and B]. In both high-grade samples, we observe CTGF^+^LRP6^+^ colocalization in malignant cells (fig. S6, C and D). The colocalization was observed in well-to-moderately differentiated regions of these high-grade tumors. These observations are consistent with our inference that CTGF:LRP6 interactions occur in regions of the tumor that may be becoming primed for more aggressive tumor growth. We observed in one of the high-grade samples distinctive CTGF^+^LRP6^−^ malignant cells (arrow 4 in Fig. 5D, ii) suggestive of M(2) subtype from the scRNA-seq analysis.

## DISCUSSION

REMI is a novel algorithm that uses graph-based approaches to generate a global cell-cell cross-talk network by estimating the conditional dependency of LR pairs on high-dimensional data of small sample size. As opposed to current methods, REMI captures the complexity of multicellular interactions by accounting for the potential confounding effects that LR pairs may have upon one another. REMI offers the main advantage of identifying codependent interactions on a global scale with small sample size by leveraging previous knowledge, network analysis, and sparsity principles. We demonstrated REMI’s performance on a large transcriptomic dataset from TCGA, showing that estimating the inverse covariance matrix using REMI is robust. We measured REMI’s performance across varying sample sizes and found that REMI’s specificity decreased less than its sensitivity relative to GLasso. REMI also outperformed existing cell-cell interaction inference approaches. The difference in performance metrics can be explained by the difference in the assumptions underlying each algorithm. CellphoneDB v2.0 predicts LR pairs on the basis of the mean expression values, as higher LR genes are more likely to be expressed and interacting. NicheNet relies on both literature-derived models and user-defined inputs to specify downstream-activated genes. Instead, REMI focuses on capturing the linear transcriptional relationship between a ligand and a receptor, which is not as well captured in the other methods. Overall, REMI’s higher specificity is favorable when choosing interactions for experimental validation to reduce the risk of false positives.

REMI is a modularized algorithm that allows investigators to adapt each module to best fit their biological question. In step 1, we used the FANTOM5 database as our reference for literature-supported LR pairs, but the database can be substituted or complemented with any other LR databases. In step 2, we used the Louvain community detection algorithm, but this can be substituted for the investigator’s clustering algorithm of choice. REMI’s community detection step is hierarchical, which allows it to accommodate high-dimensional datasets while retaining high specificity. Thus, it can be applied to the many recent single-cell studies that have revealed substantial intratumoral heterogeneity. Last, REMI was applied to transcriptomics data, but it can be generalized when applied to other -omics data as well.

We applied REMI to an LUAD bulk flow-sorted RNA-seq dataset composed of four broad cell types (malignant, immune, fibroblast, and endothelial) to assemble the REMI-LUAD microenvironment interactome. We showed that REMI-LUAD captured tumor-stroma– and tumor-stroma-immune–specific communities that were enriched for ECM remodeling interactions and highlighted the prevalence of tumor-stroma–specific interactions. To increase the cell type resolution of REMI-reconstructed networks, we projected a publicly available scRNA-seq data onto REMI-LUAD to construct scREMI-LUAD and uncovered potential interactions associated with LUAD progression. Because of the increased granularity of the interactions, we observed that previously labeled autocrine signaling interactions within REMI-LUAD became paracrine signaling interactions between cell subtypes in scREMI-LUAD. This observation suggests that the currently documented autocrine signaling interactions may be involved in paracrine signaling between different cell states for a given cell type.

To infer a cancer progression signature within scREMI-LUAD, we calculated the prognosis of each cell subtype according to its ligand expression. We then applied unsupervised analysis of prognostic-specific cellular cross-talk signatures and ordered interactions on the basis of prognosis as a proxy to tumor progression. Interactions among good-prognostic cell subtypes consisted of antitumoral or preinvasive activities, whereas interactions among poor-prognostic cell subtypes were involved in more invasive properties. We reasoned that mixed-prognostic interactions may be associated with progression from a less to more invasive TME.

We noted one mixed-prognostic interaction, namely, between CTGF and LRP6 among malignant subpopulations, and experimentally verified the presence versus the absence of colocalization of CTGF and LRP6 in high- versus low-grade primary LUAD samples. More specifically, we observed CTGF and LRP6 colocalization in well-to-moderately differentiated malignant regions of high-grade primary LUAD samples and did not observe their colocalization in low-grade tumors. These findings, while preliminary, are consistent with our inferences that CTGF and LRP6 interactions may be priming the tumor toward a more aggressive phenotype. This work suggests that anti-CTGF (*29*, *30*) therapy to inhibit progression in LUAD warrants consideration. We did not observe evidence of CTGF expression by fibroblasts interacting with LRP6 on malignant cells, which was another predicted interaction in scREMI-LUAD. This may be attributed to the low amounts of fibroblasts seen in our tissue specimens. We also did not have access to tissue blocks that captured more poorly differentiated malignant regions of the high-grade cases for further depiction of the CTGF^+^ and LRP6^+^ colocalization. Therefore, both scenarios warrant further experimental investigation. More generally, this work suggests that cell subtypes with prognostically relevant cross-talk warrant further investigation as they may be associated with disease progression.

While REMI can identify LR pairs with high specificity, it has limitations. First, the utility of REMI may be limited by the assumption that interacting ligands and receptors are correlated. By leveraging these correlations between LR genes representative of LR pairs, interactions that involve secretory molecules, such as chemokines that are typically secreted by immune cells, may not be captured well by REMI because chemokines can affect distant tissue sites. Second, REMI does not yet account for large protein complexes. One way to potentially address this issue is to regularize edges in the resulting interactome between receptors in the same protein complex along with the LR pairs in the community. Third, REMI does not account for potential external influences on LR pairs, such as spatial or temporal considerations. This may be remedied by implementing a weighted graphical Gaussian model instead. A weighted approach may also allow users to capture LR affinity or downstream signaling effects. Last, the significance test is designed for each community because the edge permutations change the communities identified by REMI. This stochasticity is difficult to capture in REMI’s current statistical test. Calculating *P* values for all edges within the network is also time intensive. Further work needs to be done to implement a fast interactome-wide hypothesis test.

In summary, we developed REMI and demonstrated the benefit of using its networks as a discovery tool. REMI provides a novel way to infer global cellular cross-talk for subsequent functional validation. We anticipate that REMI can be applied to many more tissue microenvironments to better understand normal and diseased processes and identify more precise therapeutics.

## MATERIALS AND METHODS

### Bulk flow-sorted RNA-seq data

We used publicly available bulk flow-sorted RNA-seq data (GSE111907) that had 17 primary LUAD tumor samples that, using flow cytometry, were sorted into the four cell types of interest: immune, endothelial, fibroblast, and malignant cancerous cells using the markers CD45^+^/EpCAM^−^, CD31^+^/CD45^−^/EpCAM^−^, CD10^+^/EpCAM^−^/CD45^−^/CD31^−^, and epithelial cellular adhesion molecule (EpCAM)/CD45^−^, respectively. Patients without data from all four cell types were removed. The data included bulk RNA-seq measurements for each cell type defined as *X* = {*X*_*c*1_, *X*_*c*2_, *X*_*c*3_, *X_cn_*}. The TPM (transcripts per million) values were log transformed [log_2_(TPM + 1)] and standardized to 0 mean and 1 SD.

### Publicly available databases

#### FANTOM5 database

We obtained a total of 1904 validated LR pairs (650 ligands and 594 receptors) from the FANTOM5 database (*15*). The database extracted known LR pairs from the Database of Ligand-Receptor Partners (DLRP), IUPHAR (International Union of Basic and Clinical Pharmacology), and the Human Plasma Membrane Receptome. We filtered for LR pairs that had literature-supported evidence and were experimentally validated. We included 12 more literature-supported experimentally validated LR pairs not found in the database: PDL1/PD-1, PD-2/PDL1, CD80/CTLA4, CD80/CD28, CD86/CD28, GREM1/KDR, PDL2/PD-1, NECTIN2/CD226, NECTIN2/TIGHT, PVR/TIGHT, SIGLEC1/SPN, and CTGF/TNFRSF1A.

#### KEGG database

From the KEGG database, we downloaded all the pathway genes from the Environmental Information Processing category. This includes signaling pathways associated with membrane transport, signal transduction, and signaling molecules and interactions. Genes from the phosphotransferase system and bacterial secretion system were excluded.

#### BioGRID database

We downloaded the *Homo sapiens* proteins from BioGRID and their interaction network. Only protein-protein interactions that were experimentally validated were used. We removed proteins that were found in the publicly available database CRAPome (*36*). The remaining BioGRID network has 18,074 proteins and 176,120 interactions.

### LTMI reconstruction

For the LTMI reconstruction, the log-transformed bulk flow-sorted RNA-seq data from Gentles *et al.* were split into patients with LUAD and those with lung squamous cell carcinoma. Using only the patients with LUAD (*n* = 17), LR genes were filtered by TPM > 10. A graph was constructed by creating a node for every LR gene that passed the filtering criteria, and an edge was drawn if it was a known pair according to the FANTOM5 database. This was cross-referenced with the LTMI built on all samples. The igraph R package was used to construct and illustrate the graph. We also used igraph to identify components in the network. To calculate partial correlation for the LTMI subgraph, we used the following equation

$$r_{\text{ABC}}=\frac{r_{\text{AB}}-r_{\text{AC}}r_{\text{BC}}}{\sqrt{\left(1-r_{\text{AC}}^{2})(1-r_{\text{BC}}^{2}\right)}}$$

This equation was extended to capture all combinations of gene interactions within the subgraph. Correlation was measured using the gene expression dataset.

### REMI algorithm

(i) Build weighted bipartite LR network. On the basis of a user-defined LR list, an LR network is created. The default LR list is obtained from the FANTOM5 database. Cell types from the input dataset are denoted as *c*_1_, *c*_2_, …, *c_n_* ∈ *X*. *x_c_* represents all ligands found in cell type *c* (*x*_1_, *x*_2_, …, *x_a_* ∈ *X_c_*). *y_c_* represents all receptors found in cell type *c* (*y*_1_, *y*_2_, …, *y_b_* ∈ *X_c_*). Given dataset *X*, we represent all potential LR pairs interacting between all cell types in the undirected network *G*_LR_ = (*V*_1_ = *V_x_* ∩ *V_y_*, *E*_1_), where *V_x_* = {*x* : *x*_*c*_1__, *x*_*c*_2__, …, *x_c_n__*} and *V_y_* = {*y* : *y*_*c*_1__, *y*_*c*_2__, …, *y_c_n__*}. An edge represents a relationship between ligand and its cognate receptor denoted as *E*_1_ = {(*x*, *y*) : *x* ∈ *V_x_*, *y* ∈ *V_y_*, (*x*, *y*) ∈ *N*_FANTOM5_}. Edge weight *w_ij_* in *G*_LR_ is the Pearson correlation between *x_i_* and *y_j_* calculated using the gene expression data.

(ii) Detect cognate LR communities. Communities (*C_k_*) are identified in *G*_LR_ using Louvain community detection algorithm. The algorithm identifies clusters of nodes, called communities, that are optimized for maximum modularity (*Q*) within the network

$$Q=\frac{1}{2m}\sum_{\mathrm{vw}}[A_{\mathrm{vw}}-\frac{k_{v}k_{w}}{2m}]\delta (c_{v},c_{w})$$

*A_vw_* is the Spearman correlation between nodes *v* and *w*. *m* is the number of edges. *k* is the degree of node. δ is the Kronecker delta piecewise function indicating whether node *v* or *w* is in the same community. *c* is an indicator function for whether a node is in a community. The measurement ensures that the communities detected are data driven and not based on chance. *G*_LR_ can contain disjoint components because some nodes were removed because of their low expression within the dataset. Disconnected small components are referred to as a community. If the number of nodes within *C_k_* is greater than the sample size, then we perform hierarchical community detection using Louvain to further reduce the density of the communities until the number of nodes in every community is less than or equal to the sample size. We then iterate between every pairwise combination of communities and create a pseudocommunity or BC. The BCs contain nodes that have an edge between two communities.

(iii) Remove conditionally independent LR pairs. According to the Hammersley-Clifford theorem, an inverse covariance matrix, or partial correlation, can be used to indicate relationships between nodes. A zero in an inverse covariance matrix indicates conditional independence between the two variables in the model given all others. The algorithm GLasso uses this concept and estimates the inverse covariance matrix that best fits the observations in a multivariate Gaussian distributed dataset (*17*). A *l*1 penalty is added to the log-likelihood of a Gaussian Markov field to add data-driven sparsity to the model. Here is the optimization function

$$\text{log}(\text{detΘ}-\text{tr}(S\Theta )-\lambda {||w*\Theta ||}_{1})$$

In this equation, Θ = σ^−1^ and *S* are the empirical covariance matrix. *w* represents the weighted adjacency matrix. tr is the trace of the matrix or the sum of the diagonal. ∣∣Θ∣∣_1_ is the *l*1 penalization of the inverse covariance matrix. When λ increases, the network becomes sparser. When λ is zero, the resulting network is equivalent to a partial correlation network (*17*). We use GLasso from the glasso R package to measure the conditional dependence between two connected nodes while controlling for the effect of all other nodes for each community detected. Because the communities identified within the largest network component are not disjoint clusters, we also created graphical models using the nodes and edges BCs. These BCs only include nodes that have edges BCs and excludes nodes that do not. Communities with one node were not included.

The GLasso similarity matrix can be represented as a fully connected LR community graph. This includes all potential edges between LRs, ligand and ligand (LL), and receptor and receptor (RR). We simplify the LR prediction problem by focusing on only LR interactions and make the assumption that the underlying network that represents the data only contains edges between LRs. LL and RR edges within the *G*_LR_ communities are penalized and set to zero in the weighted adjacency matrix, indicating conditional independence. The penalized weighted matrix is defined as

$$w_{\mathrm{ij}}=\{\begin{array}{c} \lambda ,\text{if}\;(i,j)\in C_{k}\\ 0,\text{if}(i)\in C_{k},(j)\in C_{k\prime },k!=k\prime \end{array}$$

The λ tuning parameter in the penalized log-likelihood equation is chosen by iterating through 20 values logarithmically spaced between lambda values 0.01 to 0.9. The optimal λ is the one that best minimizes the Bayesian information criterion. The accuracy of the GLasso method is dependent on the number of features in the network.

(iv) Reconstruct communities into predicted interactome. The final reconstructed LR network includes edges between nodes that have a weight larger than zero.

(v) Significance test for *P* value. We provide an optional additional tool to calculate *P* values for an edge in a community. *P* values are calculated by measuring the occurrence of a test statistic compared with its null. In the REMI algorithm, an LR pair is a subset of a test statistic, the regularized covariance matrix. Therefore, our significance test measures the conditional probability that an LR pair is conditionally dependent within its REMI community given added randomization. First, we sample from a uniform distribution of correlation values and replace the correlation value of the LR pair of interest. Then, REMI is rerun 1000 times to generate a distribution of predicted scores for the LR pair. A regression tree is fit on the sampled correlation values to predict binary REMI scores. A null distribution is lastly generated by simulating a null training dataset by creating a Gaussian distribution (*G*) and training it on the regression tree to predict a null set of outcomes (*w*). *w* is multiplied by the Wishart distribution to create a null distribution that represents the conditional test statistic (*37*). The *P* value is then calculated by using the LR pair’s data-derived correlation value (*R*[*i*, *j*]). The first equation represents the one-sided *P* value, and the second equation represents the two-sided *P* value

$$p=\frac{\sum_{n}w*(G>R[i,j])}{\sum_{n}w}$$

p=2*min(p,1−p)

### Validation dataset

We tested the method on a bulk RNA-seq dataset downloaded from the publicly available database, TCGA. The non–small cell lung carcinoma (NSCLC) RNA-seq dataset contains bulk RNA-seq data for 1013 patients with NSCLC. This includes 584 patients with LUAD and 429 patients with lung squamous cell carcinoma. The data were log transformed and scaled to a mean of 0 and a variance of 1.

### Simulations

The TCGA-LUAD GLasso interactome was generated by running GLasso interactome (GLasso on *G*_LR_) using LR genes found in the TCGA NSCLC bulk RNA-seq dataset. In the resulting network (*V* = 893 and *E* = 1331), a true-positive edge is an edge with weight greater than zero. All other edges are true-negative edges. Five cohort sizes (*n* = 10, 25, 50, 100, and 2000) were sampled 50 times each from the whole TCGA NSCLC cohort to simulate various cohort sizes. REMI was run on each sampled cohort dataset using default parameters and unfiltered list of LR genes. GLasso was also performed on each sampled cohort dataset with restrictions on the LL and RR edges. Performance metrics were calculated by setting the reference “gold standard” interactome as the TCGA-LUAD GLasso interactome. Correlation values of the LR pairs were used as the cutoff for REMI’s AUC calculations. The same analysis was performed by separating WCs’ and BCs’ predictions and calculating their performance metrics.

### Comparison against other methods

#### NicheNet

We ran NicheNet on TCGA NSCLC bulk RNA-seq dataset using the same filtered genelist as the ones used in TCGA simulations described above. The background gene set used included the genes found in the ligand target matrix of the receiving cell. The LR list was replaced with the FANTOM5 list we generated. For TCGA data, we treated the bulk dataset as an autocrine signaling. We ran NicheNet on every autocrine and paracrine combination of cell types. We used the best ligand filter of Pearson >0.

#### NATMI

We used both log-transformed and scaled log-transformed TCGA RNA-seq dataset as input and set all samples as one cell type. Default parameters were used, and edges were filtered on the basis of specificity score.

#### CellphoneDB

We used the log-transformed TCGA RNA-seq dataset as input for the method. We used the database provided and filtered for significant LR pairs based on the significant means score.

#### CCCExplorer

We ran the CCCExplorer on TCGA NSCLC bulk RNA-seq dataset and bulk flow-sorted RNA-seq dataset. To identify differentially expressed gene (DEG) ligands for the input, we used samr to identify DEGs (*38*). For TCGA, we identified DEG between healthy patients and patients with lung cancer. For the bulk flow-sorted cohort, we identified DEG between the sending cell and receiving cell. The DEG cutoffs used were logFC >1.2 and *q* < 0.05. The same DEG input from NicheNet was used. We calculated cell-cell interactions for every pairwise paracrine and autocrine combination.

### REMI analysis in the LUAD dataset

For the REMI interactome construction, the log-normalized gene expression measurements were binned into three evenly split discretized groups for each cell type. Genes with expression levels falling within the first group, representing low expressed genes, were removed. The dispersion ratio (variance/mean) was calculated for each gene and normalized within each bin. Genes in the first bin, which includes genes with negative and fairly low expression levels, were removed. The data were scaled after filtering, and the filtered dataset was used to identify LR genes. The scaled unfiltered gene expression dataset was used for all other analyses. All other parameters in REMI were set to default.

### Gene enrichment analysis

The clusterProfiler R package was used to perform gene enrichment analysis. The gene lists were filtered for the databases: Hallmark, BIOCARTA, and REACTOME. Enrichments with adjusted *P* < 0.05 were used for analysis.

### scRNA-seq analysis

#### Data

We used publicly available scRNA-seq datasets E-MTAB-6149 and E-MTAB-6653 generated by Lambrecht *et al.* (*21*). The droplet-based scRNA-seq (10X genomics) datasets had two patients with squamous cell carcinoma, two patients with adenocarcinoma, and one patient with NSCLC. We used the Seurat V3 R package to process the patients with LUAD and used predefined cell type labels defined by the authors (*39*).

#### Preprocessing

In cells with more than 10% mitochondria content, over 6000 and under 100 features were filtered out. We used the top 30 principal components and the IntegrateData function in Seurat to correct for batch effect between the patients. Malignant, fibroblast, and endothelial cells were reclustered using Louvain clustering on a Shared Nearest Neighbor graph with 10 principal components using 0.2, 0.2, and 0.1 resolutions, respectively. DEGs across all cell types and within clusters were calculated using model-based analysis of single cell transcriptomics (MAST) with default parameters and normalized counts (averaged log_2_FC > 0.25; Bonferroni-corrected *P* < 0.05) (*40*). Visualizations are shown using uniform manifold approximation and projection (UMAP) (30 principal components). PHATE (potential of heat diffusion for affinity-based transition embedding) was used to infer the underlying hierarchical manifold in each cell type using default parameters (*41*). Each cell type population was imputed using adaptively-thresholded low rank approximation (ALRA) after identifying subpopulations (*42*).

### Deconvolving interactome

LR genes in the REMI-LUAD interactome were filtered on the basis of whether they were expressed in their respective cell type (average expression, >0.4). The cell type metadata associated with each node in the interactome was relabeled using the subpopulation in which the ligand or receptor was found in. The cell type must be the same between bulk and single-cell data. Nodes with genes that were found across multiple subpopulations within the same cell type were duplicated, and an edge drawn to its respective predicted cognate pair.

### Measuring prognosis of subpopulations

Bulk LUAD-specific prognosis scores were downloaded from the PRECOG metaZ database. Then, the normalized log-transformed average expression level for the ligands within each subpopulation was calculated using the ALRA imputed gene expression data. Each subpopulation was imputed independently. For each subpopulation, ligands that were not DE, as calculated by MAST in a prior step, were removed. DE ligand cognate receptors with an average scaled expression less than 0 were removed. Subpopulations with less than three DE ligands predicted to be secreted were also removed from the calculations to increase the power of the calculation. In the remaining list of LR pairs, we then multiplied the DE ligand’s averaged expression level by its PRECOG score [$a=\sum_{n}e_{i}^{L}*\text{PRECOG}(L)$]. The total gene expression values of ligands ($b=\sum_{n}e_{i}^{L}$) were aggregated, and the prognosis of the subpopulation by *a*/*b* was calculated. *n* is the total number of cells in a subtype. *i* is the gene index. *L* is the ligand of interest. Additional filtering of the LR pairs was then applied to duplicate LR pairs that have the same genes but different sending and receiving cells. The pair with the closest PRECOG score between sending and receiving cells was selected.

### IF and H&E staining

Clinical aspects of this study were approved by the Stanford Institutional Review Board (IRB) in accordance with the Declaration of Helsinki guidelines for the ethical conduct of research. All patients involved provided a written informed consent. Collection and use of human tissues were approved and in compliance with data protection regulations regarding patient confidentiality (IRB protocol no. 15166). Following surgical resection of primary tumors from patients at Stanford Hospital, LUAD specimens were immediately embedded in optimal cutting temperature (OCT) compound. Fresh-frozen human LUAD tissues were serially sectioned at a thickness of 8 μm and mounted on poly-l-lysine–coated coverslips. One section was successively fixed in 95% ethanol and 10% formalin for 10 min each and subjected to progressive H&E staining following the standard protocol described (*43*) and reviewed by a board-certified pathologist (M.E.O.). For IF analysis, sections were fixed in acetone for 10 min at room temperature (RT) and then washed twice with phosphate-buffered saline (PBS). Fluorophore bleaching was performed at RT for 90 min to reduce autofluorescence by immersing the fixed coverslip in freshly prepared bleaching solution [4.5% (w/v) H_2_O_2_ and 20 mM NaOH in PBS] and illuminating the container between two light-emitting diode (LED) light panels, followed by four washes with PBS to remove bleaching solution (*44*). The pretreated coverslips were blocked with 1% horse serum in PBS for 30 min and incubated with primary antibodies in incubation solution (1% bovine serum albumin, 1% normal donkey serum, 0.3% Triton X-100, and 0.01% sodium azide in PBS) in a humidified chamber overnight at 4°C. After three washes in PBS for 15 min per wash, the coverslips were incubated with secondary antibodies in incubation solution for 1 hour at RT in dark. The coverslips were intensively washed with PBS and then stained with DRAQ5 (Thermo Fisher Scientific) at 1:1000 in PBS for 10 min. The sections were observed under the BZ-X800 fluorescence microscope (Keyence, IL, USA). Antibodies used in the study include anti-CTGF (ab6992, Abcam), anti-LRP6 (MAB1505, R&D System), anti-EpCAM (no. 2929, Cell Signaling Technology), anti-pan Cytokeratin (ab86734, Invitrogen), Cy3-conjugated goat anti-rabbit immunoglobulin G (IgG) (ab6939, Abcam), goat anti-mIgG1 Alexa Fluor 488 (A-21121, Invitrogen), and donkey anti-mouse IgG H&L Alexa Fluor 488 (ab150105, Abcam) antibodies.

### Downstream influence score

An undirected downstream signaling pathway network *G*_ds_ = (*V*_ds_, *E*_ds_) is created for each cell type in the dataset. Each *G*_ds_ consists of approximately 1000 nodes representing signal transduction pathway genes (*N*_KEGG_) downloaded from the publicly available database, KEGG (*V*_ds_ = {*x* : *x* ∈ *N*_KEGG_}) (*34*). An edge between two nodes represents a physical protein-protein interaction between the nodes, as indicated in the database BioGRID (*E*_ds_ = {(*x_i_*, *x_j_*) ∈ *N*_BioGRID_}) (*33*). The edge weight in the network is set as the Pearson correlation between the two gene nodes calculated from the gene expression data from the cell type of interest. For each *G*_ds_ per cell type, we calculate the eigenvector centrality for each node. Eigenvector centrality is a centrality measurement of a node *x_i_* that is relational to the sum of importance of its neighbor nodes: $x_{i}=\frac{1}{\lambda }\sum_{j\in G}w_{i,j}x_{j}$, where *w*_*i*, *j*_ is the edge weight between nodes *x_i_* and *x_j_*. The centrality measurement for each receptor within *G*_ds_ is the receptor’s influence score.

## Notes

View/request a protocol for this paper from *Bio-protocol*.
