# Supplementary material for: Reconstructing codependent cellular cross-talk in lung adenocarcinoma using REMI
Source: Sci Adv. 2022 Mar 18;8(11):eabi4757. doi: 10.1126/sciadv.abi4757 (PMC8932661; doi:10.1126/sciadv.abi4757)
Supplement: Supplementary file 1 — Figs. S1 to S6 [file sciadv.abi4757_sm.pdf]

Science Advances
AAAS

Supplementary Materials for

Reconstructing codependent cellular cross-talk in lung adenocarcinoma using REMI

Alice Yu, Yuanyuan Li, Irene Li, Michael G. Ozawa, Christine Yeh, Aaron E. Chiou, Winston L. Trope, Jonathan Taylor, Joseph Shrager, Sylvia K. Plevritis*

*Corresponding author. Email: sylvia.plevritis@stanford.edu

Published 18 March 2022, *Sci. Adv.* **8**, eabi4757 (2022)
DOI: 10.1126/sciadv.abi4757

The PDF file includes:

Figs. S1 to S6
Legends for data files S1 to S6

Other Supplementary Material for this manuscript includes the following:

Data files S1 to S6

Supplementary Figures

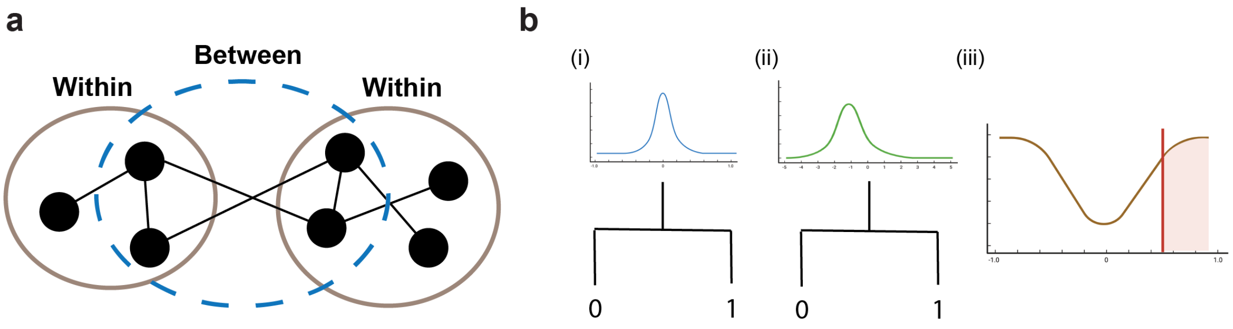

Fig. S1. Diagram of REMI intermediate steps. (a) Schematic of an overlapping community that captures edges between communities. **(b)** Diagram of steps to calculate p-value for a given ligand-receptor edge. (i) Sampling correlation values to replace edge of interest and creating a tree to predict whether edge appears or not in REMI network. (ii) Sampling from null inverse Wilshart distribution and using classification trees to predict whether edge is present or not (iii) Calculating number of randomly sampled edge values with higher predicted correlation than correlation from true data to estimate two-sided p-value.

a

	Within		Between	
Cohort Size (*n*)	Sensitivity	Specificity	Sensitivity	Specificity
**15**	0.75	0.45	0.45	0.76
**25**	0.78	0.48	0.49	0.78
**50**	0.84	0.50	0.62	0.75
**100**	0.89	0.55	0.76	0.73
**200**	0.93	0.63	0.88	0.74

b

	REMI	
Community Size (*n* = Sample Size)	Sensitivity	Specificity
**2*n***	0.66	0.52
**5*n***	0.68	0.47
**10*n***	0.69	0.47
**20*n***	0.69	0.47
**50*n***	0.78	0.33

Fig. S2. TCGA simulations to test robustness of community parameters. (a) Performance within and between communities in the TCGA simulations compared to population-level TCGA-LUAD GLasso interactome. Accuracy metrics are averaged across the 50 sampled datasets per cohort size. **(b)** Performance of REMI when the number of nodes is increased within the community from 2n to 50n. Accuracy metrics are averaged across the 50 sampled datasets per cohort size.

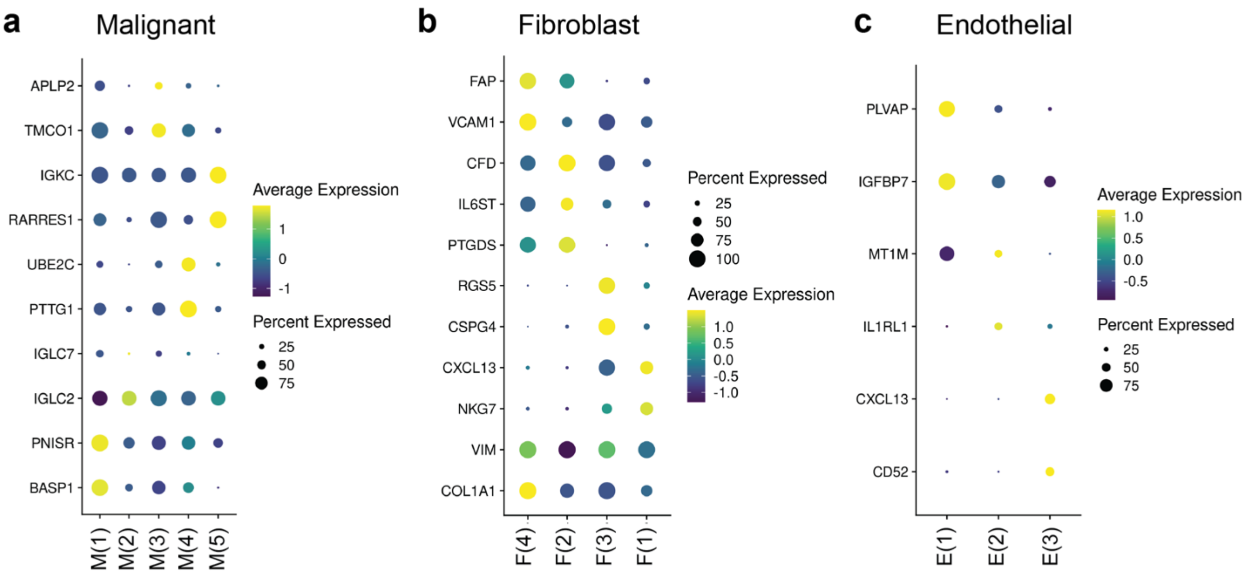

Fig. S3. Defining TME subpopulation characteristics. (a) Averaged scaled expression levels of top expressed differentially expressed (DE) markers for each malignant subpopulation **(b)** Averaged scaled expression levels of top expressed DE markers for each fibroblast subpopulation **(c)** Averaged scaled expression levels of top expressed DE markers for each endothelial subpopulation

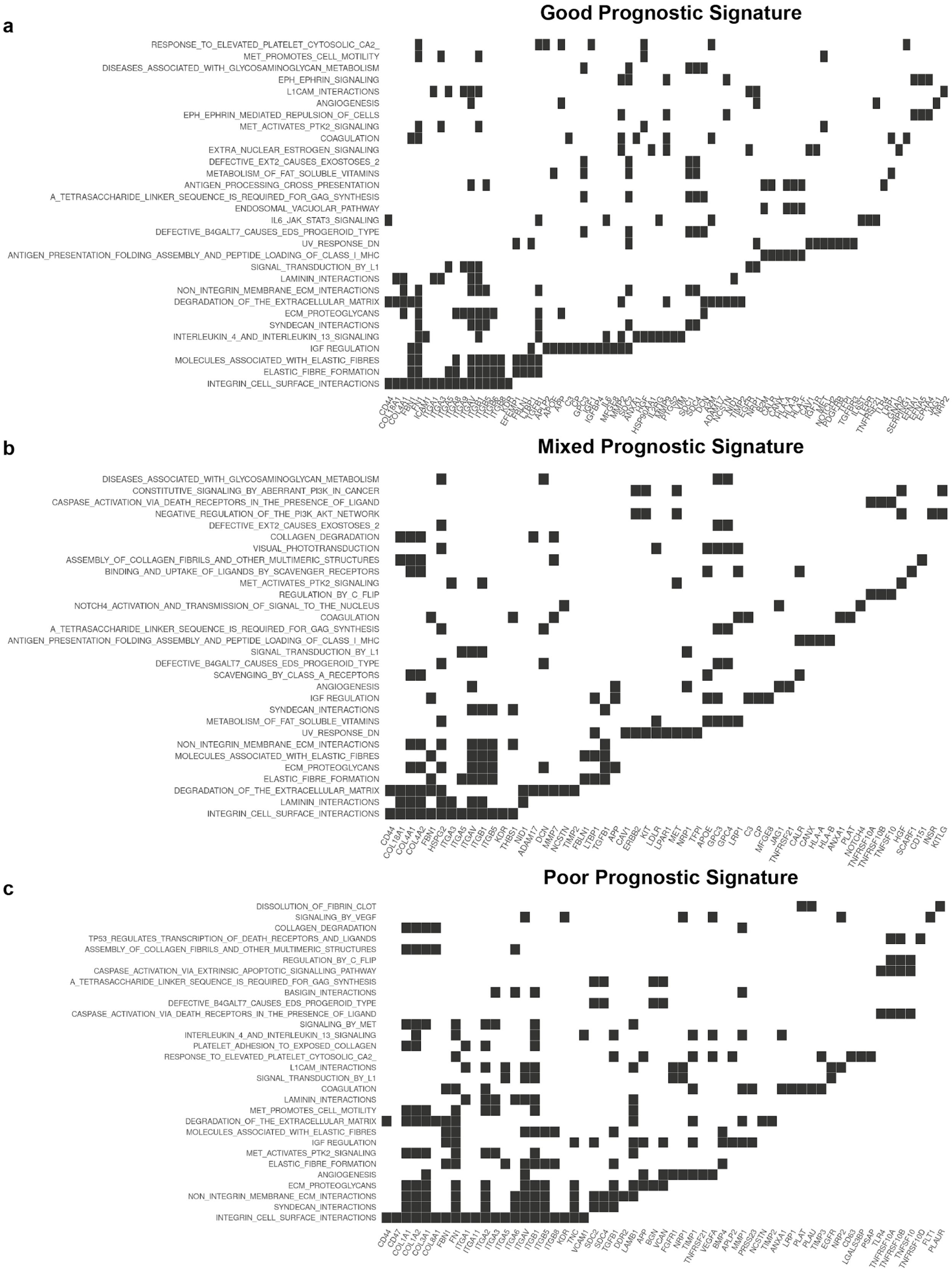

a
Good Prognostic Signature
RESPONSE_TO_ELEVATED_PLATELET_CYTOSOLIC_CA2_
MET_PROMOTES_CELL_MOTILITY
DISEASES_ASSOCIATED_WITH_GLYCOSAMINOGLYCAN_METABOLISM
EPH_EPHRIN_SIGNALING
L1CAM_INTERACTIONS
ANGIOGENESIS
EPH_EPHRIN_MEDIATED_REPULSION_OF_CELLS
MET_ACTIVATES_PTK2_SIGNALING
COAGULATION
EXTRA_NUCLEAR_ESTROGEN_SIGNALING
DEFECTIVE_EXT2_CAUSES_EXOSTOSES_2
METABOLISM_OF_FAT_SOLUBLE_VITAMINS
ANTIGEN_PROCESSING_CROSS_PRESENTATION
A_TETRASACCHARIDE_LINKER_SEQUENCE_IS_REQUIRED_FOR_GAG_SYNTHESIS
ENDOSOMAL_VACUOLAR_PATHWAY
IL6_JAK_STAT3_SIGNALING
DEFECTIVE_B4GALT7_CAUSES_EDS_PROGEROID_TYPE
UV_RESPONSE_DN
ANTIGEN_PRESENTATION_FOLDING_ASSEMBLY_AND_PEPTIDE_LOADING_OF_CLASS_I_MHC
SIGNAL_TRANSDUCTION_BY_L1
LAMININ_INTERACTIONS
NON_INTEGRIN_MEMBRANE_ECM_INTERACTIONS
DEGRADATION_OF_THE_EXTRACELLULAR_MATRIX
ECM_PROTEOGLYCANS
SYNDECAN_INTERACTIONS
INTERLEUKIN_4_AND_INTERLEUKIN_13_SIGNALING
IGF REGULATION
MOLECULES_ASSOCIATED_WITH_ELASTIC_FIBRES
ELASTIC_FIBRE_FORMATION
INTEGRIN_CELL_SURFACE_INTERACTIONS
b
Mixed Prognostic Signature
DISEASES_ASSOCIATED_WITH_GLYCOSAMINOGLYCAN_METABOLISM
CONSTITUTIVE_SIGNALING_BY_ABERRANT_PI3K_IN_CANCER
CASPASE_ACTIVATION_VIA_DEATH_RECEPTORS_IN_THE_PRESENCE_OF_LIGAND
NEGATIVE_REGULATION_OF_THE_PI3K_AKT_NETWORK
DEFECTIVE_EXT2_CAUSES_EXOSTOSES_2
COLLAGEN_DEGRADATION
VISUAL_PHOTOTRANSDUCTION
ASSEMBLY_OF_COLLAGEN_FIBRILS_AND_OTHER_MULTIMERIC_STRUCTURES
BINDING_AND_UPTAKE_OF_LIGANDS_BY_SCAVENGER_RECEPTORS
MET_ACTIVATES_PTK2_SIGNALING
REGULATION_BY_C_FLIP
NOTCH4_ACTIVATION_AND_TRANSMISSION_OF_SIGNAL_TO_THE_NUCLEUS
COAGULATION
A_TETRASACCHARIDE_LINKER_SEQUENCE_IS_REQUIRED_FOR_GAG_SYNTHESIS
ANTIGEN_PRESENTATION_FOLDING_ASSEMBLY_AND_PEPTIDE_LOADING_OF_CLASS_I_MHC
SIGNAL_TRANSDUCTION_BY_L1
DEFECTIVE_B4GALT7_CAUSES_EDS_PROGEROID_TYPE
SCAVENGING_BY_CLASS_A_RECEPTORS
ANGIOGENESIS
IGF REGULATION
SYNDECAN_INTERACTIONS
METABOLISM_OF_FAT_SOLUBLE_VITAMINS
UV_RESPONSE_DN
NON_INTEGRIN_MEMBRANE_ECM_INTERACTIONS
MOLECULES_ASSOCIATED_WITH_ELASTIC_FIBRES
ECM_PROTEOGLYCANS
ELASTIC_FIBRE_FORMATION
DEGRADATION_OF_THE_EXTRACELLULAR_MATRIX
LAMININ_INTERACTIONS
INTEGRIN_CELL_SURFACE_INTERACTIONS
CD44
COL18A1
COL4A1
COL4A2
FBN1
HSPG2
ITGA3
ITGA5
ITGAV
ITGB1
ITGB5
KDR
THBS1
NID1
ADAM17
DCN
MMP7
NCSTN
TIMP2
FBLN1
LTBP1
TGFB1
APP
CAV1
ERBB2
KIT
LDLR
LPAR1
MET
NRP1
TFPI
APOE
GPC3
GPC4
LRP1
C3
CP
MFGE8
JAG1
TNFRSF21
CALR
CANX
HLA-A
HLA-B
ANXA1
PLAT
NOTCH4
TNFRSF10A
TNFRSF10B
TNFSF10
HGF
SCARF1
CD151
INSR
KITLG
c
Poor Prognostic Signature
DISSOLUTION_OF_FIBRIN_CLOT
SIGNALING_BY_VEGF
COLLAGEN_DEGRADATION
TP53_REGULATES_TRANSCRIPTION_OF_DEATH_RECEPTORS_AND_LIGANDS
ASSEMBLY_OF_COLLAGEN_FIBRILS_AND_OTHER_MULTIMERIC_STRUCTURES
REGULATION_BY_C_FLIP
CASPASE_ACTIVATION_VIA_EXTRINSIC_APOPTOTIC_SIGNALLING_PATHWAY
A_TETRASACCHARIDE_LINKER_SEQUENCE_IS_REQUIRED_FOR_GAG_SYNTHESIS
BASIGIN_INTERACTIONS
DEFECTIVE_B4GALT7_CAUSES_EDS_PROGEROID_TYPE
CASPASE_ACTIVATION_VIA_DEATH_RECEPTORS_IN_THE_PRESENCE_OF_LIGAND
SIGNALING_BY_MET
INTERLEUKIN_4_AND_INTERLEUKIN_13_SIGNALING
PLATELET_ADHESION_TO_EXPOSED_COLLAGEN
RESPONSE_TO_ELEVATED_PLATELET_CYTOSOLIC_CA2_
L1CAM_INTERACTIONS
SIGNAL_TRANSDUCTION_BY_L1
COAGULATION
LAMININ_INTERACTIONS
MET_PROMOTES_CELL_MOTILITY
DEGRADATION_OF_THE_EXTRACELLULAR_MATRIX
MOLECULES_ASSOCIATED_WITH_ELASTIC_FIBRES
IGF REGULATION
MET_ACTIVATES_PTK2_SIGNALING
ELASTIC_FIBRE_FORMATION
ANGIOGENESIS
ECM_PROTEOGLYCANS
NON_INTEGRIN_MEMBRANE_ECM_INTERACTIONS
SYNDECAN_INTERACTIONS
INTEGRIN_CELL_SURFACE_INTERACTIONS
CD44
CD47
COL1A1
COL1A2
COL3A1
COL6A1
FBN1
FN1
ITGA1
ITGA11
ITGA2
ITGA3
ITGA5
ITGA6
ITGAV
ITGB1
ITGB5
ITGB8
KDR
TNC
VCAM1
SDC2
SDC4
TGFB1
DDR2
LAMB1
APP
BGN
VCAN
FGFR1
NRP1
TIMP1
TNFRSF21
VEGFA
BMP4
APLP2
MMP1
PRSS23
NCSTN
TIMP2
ANXA1
LRP1
PLAT
PLAU
TIMP3
EGFR
NRP2
CD63
LGALS3BP
PSAP
TLR4
TNFRSF10A
TNFRSF10B
TNFSF10
TNFRSF10D
FLT1
PLAUR

Fig. S4. Enrichment of prognostic signatures. (a) Enriched genesets for ligand and receptor genes in good prognostic signature (adj p-value < 0.05, max GS size = 150) **(b)** Enriched genesets for ligand and receptor genes in mixed prognostic signature (adj p-value < 0.05, max GS size = 150) **(c)** Enriched genesets for ligand and receptor genes in poor prognostic signature (adj p-value < 0.05, max GS size = 150)

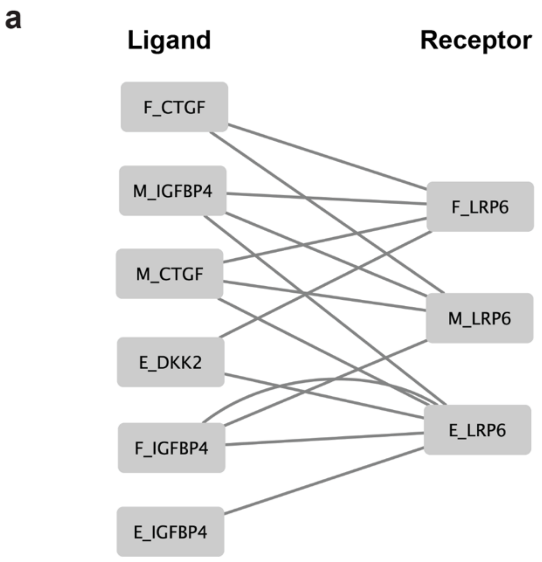

b

Ligand	Receptor	p-value
E_DKK2	E_LRP6	0.054
E_IGFBP4	E_LRP6	0.64
F_IGFBP4	E_LRP6	0.12
E_DKK2	F_LRP6	0.01
F_CTGF	F_LRP6	NA
F_IGFBP4	E_LRP6	0.12
M_CTGF	E_LRP6	0.94
M_CTGF	F_LRP6	NA
M_IGFBP4	E_LRP6	0.15
M_IGFBP4	F_LRP6	NA
F_CTGF	M_LRP6	0.059
F_IGFBP4	M_LRP6	0.13
M_CTGF	M_LRP6	0.049
M_IGFBP4	M_LRP6	0.15

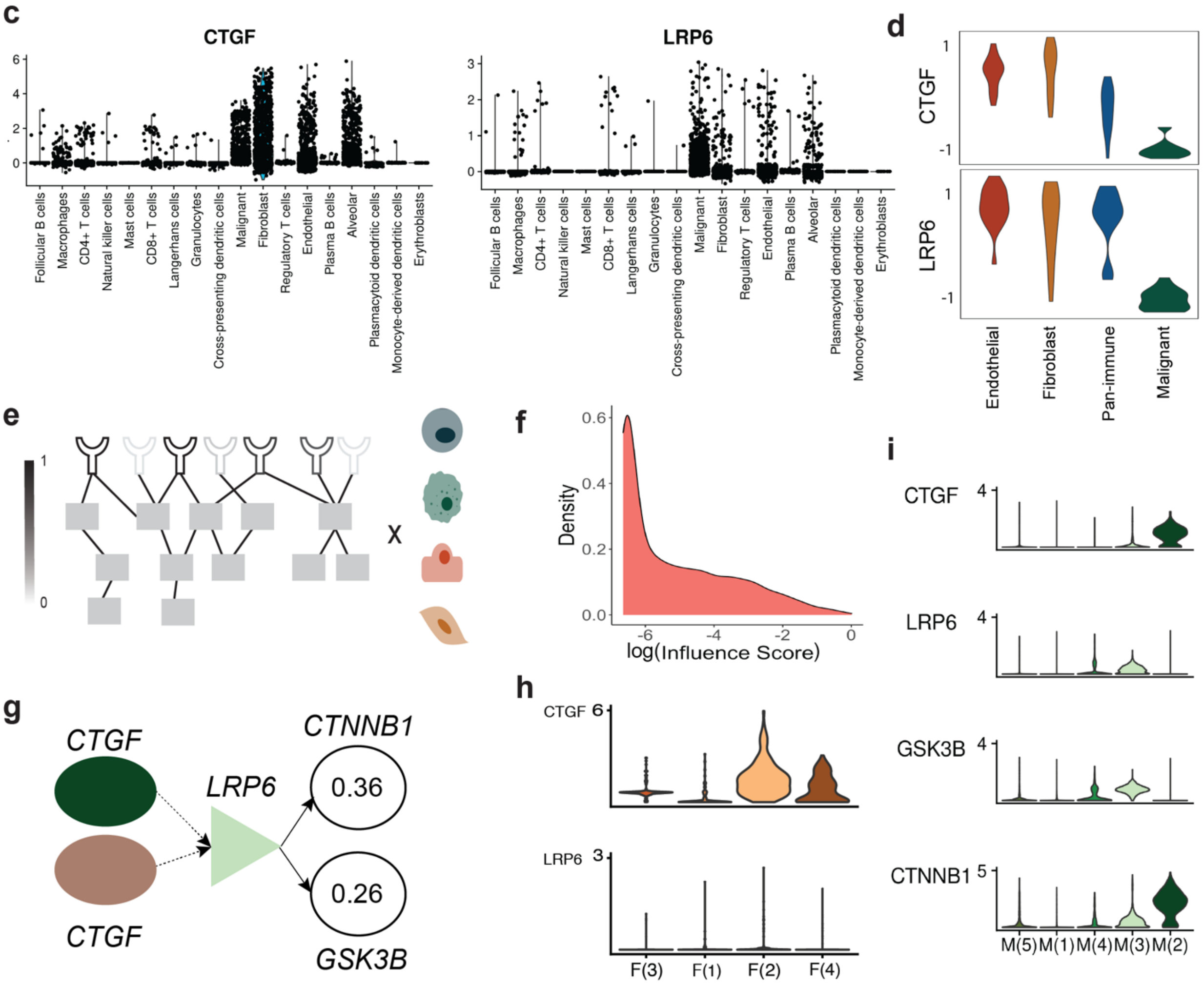

Fig. S5. CTGF scREMI community characteristics. (a) Network visualization of the predicted community network (community #50) containing CTGF expressed on malignant cells. Nodes are labeled as celltype_genename. An edge indicates a predicted LR pair. **(b)** Table of p-values for each edge. NA represents pairs where perturbing that particular edge did not lead to a converged result. **(c)** Scaled expression of *CTGF* and *LRP6* across cell types in the single-cell RNA-seq data **(d)** Scaled expression levels of *CTGF* and *LRP6* across bulk flow-sorted RNA-seq levels **(e)** Visual diagram of how to calculate downstream receptor score. A downstream signaling pathway network is built for each distinct cell type in the dataset. Eigenvector centrality is calculated for each downstream signaling gene to measure how "activated" they are within the cell-type. **(f)** Distribution of downstream centrality scores on a log scale for all four downstream signaling pathway networks. **(g)** *GSK3B* and *CTNNB1* are downstream genes from *LRP6* found in the downstream signaling networks generated by REMI. The number within the node represents the influence score, which represents how correlated the receptor is to its downstream signaling pathway genes. Color of the nodes reflect the subpopulation label. Shape of the node represents the type of gene (solid circle = ligand, solid triangle = receptor, transparent circle = downstream gene). **(h)** Scaled expression levels of *CTGF* and *LRP6* across fibroblast subpopulations. **(i)** Scaled expression levels of *CTGF*, *LRP6*, *GSK3B*, and *CTNNB1* across malignant subpopulations.

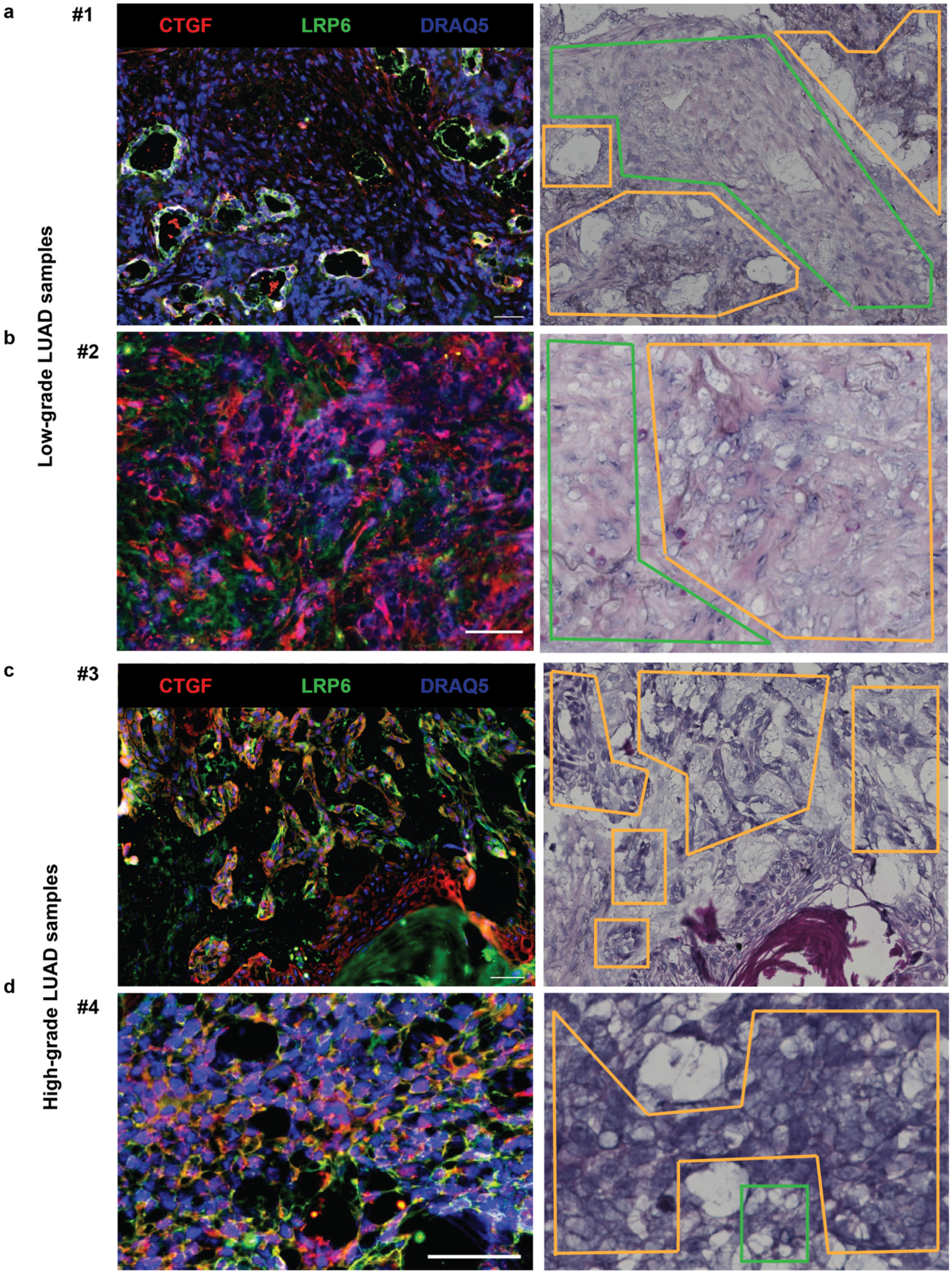

a
#1
CTGF
LRP6
DRAQ5
b
#2
Low-grade LUAD samples
c
#3
CTGF
LRP6
DRAQ5
d
#4
High-grade LUAD samples

Fig. S6. Paired immunofluorescence (IF) and H&E images of primary LUAD fresh frozen tissue samples. H&E images (right) taken after immunofluorescence (IF) staining (left). On H&E images, regions enriched with malignant versus stromal cells are encapsulated by borders colored orange versus green, respectively. **(a)** Low-grade sample from LUAD patient #1 showing expression of CTGF and LRP6 expressing cells with low to no co-localization of CTGF and LRP6. **(b)** Low-grade sample from LUAD patient #2 from our lung biobank showing expression of CTGF and LRP6 expressing cells with low to no co-localization of CTGF and LRP6. **(c)** High-grade sample from LUAD patient #3 showing malignant cells with co-expression of CTGF and LRP6 and a small group of malignant cells expressing only CTGF. **(d)** High-grade sample from LUAD patient #4 show co-expression of CTGF and LRP6 in malignant cells. IF images were stained for CTGF (red), LRP6 (green), and DRAQ5 (blue). Scale bar is 50 μM. IF images in (a) and (c) are the same as main-text Figure 5d, (i) and (ii), respectively.

Supplementary Data

Data File 1. REMI_LUAD_network.csv. Table of REMI-LUAD ligand-receptor interactions.

Data File 2. TCGA-LUAD_allsamples.csv. Table of Glasso-derived ligand-receptor interactions from the Cancer Genome Atlas (TCGA) lung adenocarcinoma (LUAD).

Data File 3. GoodPrognosis_Interactions.csv. Table of scREMI-LUAD ligand-receptor interactions associated with good prognosis.

Data File 4. MixedPrognosis_Interactions.csv. Table of scREMI-LUAD ligand-receptor interactions associated with mixed prognosis.

Data File 5. PoorPrognosis_Interactions.csv. Table of scREMI-LUAD ligand-receptor interactions associated with poor prognosis.

Data File 6. Sample_Information.docx. Clinical annotations and histopathological information of LUAD specimens
