# Supplementary material for: Reconstructing codependent cellular cross-talk in lung adenocarcinoma using REMI
Source: Sci Adv. 2022 Mar 18;8(11):eabi4757. doi: 10.1126/sciadv.abi4757 (PMC8932661; doi:10.1126/sciadv.abi4757)
Supplement: Supplementary file 2 — Data files S1 to S6 [file sciadv.abi4757_data_files_s1_to_s6.zip › sciadv.abi4757_data_file_s6.docx]

| **SPECIMEN** | **STAGE** | **TUMOR SIZE (CM)** | **DIFFERENTIATION STATUS** | **SMOKER STATUS** | **NODE STATUS** |
| --- | --- | --- | --- | --- | --- |
| 1 | 2a | Unknown | Well | Former Smoker | N0 |
| 2 | 4 | 6.0 | Moderate | Never | N0 |
| 3 | 3 | 4.8 | Moderate-poor | Never | N1 |
| 4 | 2a | Unknown | Poor | Former Smoker | N2 |

**Table 1.** ﻿**Clinical annotations and histopathological information of LUAD specimens**
